# Predicting Regulatory Product Approvals Using a Proposed Quantitative Version of FDA’s Benefit–Risk Framework to Calculate Net-Benefit Score and Benefit–Risk Ratio

**DOI:** 10.1007/s43441-020-00197-1

**Published:** 2020-07-08

**Authors:** Stephen Sun, Suzanne Heske, Melanie Mercadel, Jean Wimmer

**Affiliations:** Syneos Health, 1030 Sync Street, Morrisville, NC 27560 USA

**Keywords:** FDA, Benefit–risk framework, Net-benefit, Scoring, Risk management

## Abstract

**Background:**

Approval of regulated medical products in the USA is based upon a rigorous review of the benefits and risks as performed by the US Food and Drug Administration (FDA) staff of scientists and is summarized in a descriptive and qualitative format called the FDA’s Benefit–Risk Framework (BRF). This present method highlights the key factors in regulatory decision-making, but does not clearly define the reason for its final approval.

**Method:**

This study proposes a quantitative version of FDA’s BRF to calculate a Net-Benefit Score and a Benefit–Risk Ratio as a method to define a single-value summary of the tradeoffs between benefits and risks and allow comparisons among other products. In this retrospective review of five years of new molecular entities and new biologic (*N* = 185 products) regulatory decision-making, this proposed scoring system codifies and quantitates the information about a product’s benefits, risks, and risk management information in a format that may predict why regulated medical products are approved in the USA.

**Results:**

Simple calculation of codified benefits, risks, and risk mitigations with numerical limits is proposed to provide a repeatable process and transparency for documenting the net-benefit of regulatory product approval.

**Conclusion:**

Use of a strict process of collecting, codifying, and analyzing public information to determine a Net-Benefit score and a Benefit–Risk Ratio is possible to anticipate regulatory product approval.

## Background

US Food and Drug Administration (FDA) review and approval of market applications for regulated drugs and biologics involve a multidisciplinary team of scientific experts who make qualitative assessments from historic and study-based documents submitted by industry sponsors. The submitted data are subsequently verified and checked independently by FDA’s own reviewers. Ultimately, all relevant information is analyzed and prioritized to determine if a product should be approved as part of a assessing a product’s benefit–risk assessment [1]. As stakeholders continue to press for faster review decisions while accommodating for more complex review scenarios and technologies, e.g., emergence of new drugs and biologics, new regulatory incentive options, combination product classes, and priorities to incorporate patient inputs, a more efficient method for assessing the approvability of products will be required without compromising the scientific review process.

The basis for FDA's decision-making in the approval of regulatory products is outlined in the Benefit–Risk Framework and is simplified into a two-section summary entitled "Benefit–Risk Integrated Assessments (BRIA)" (prose) and the "Benefit–Risk Dimensions" (table) [1]. While the BRIA may provide a continuous, prose narrative, the Dimensions, commonly referred to as the benefit–risk table (BRT), does delineate the information into a structured framework of “Evidence and Uncertainties” and “Conclusions and Reasons” (columns) that is matched by the following parameters (rows): “Analysis of Condition”, “Current Treatment Options”, “Benefits”, and “Risks and Risk Management”. However, while the BRT is invaluable in providing all the key factors that contribute to a product’s regulatory approval, this method lacks a transparent measurement of too much, not enough, or how much leading to default subjective interpretation. Establishing a common numerical benefit and risk scoring standard could provide regulatory reviewers (and advisory committee members) greater consistency in decision-making across all applications with justifiable fairness and transparency and enable Sponsors optimal efficiency in targeting specific deficiencies with pinpoint deliberateness, e.g., benefit score is too low, risk score is too high, risk management is insufficient, etc. In non-regulatory settings, the award of National Institutes of Health (NIH) grants are based upon the numerical scoring of the criterion merits of an application by its reviewers on a 9-point rating scale [2]. Consequently, if this information could be translated into quantitative value(s), calculation comparisons and pattern assessments between products and time periods could be possible [3].

Numerous mathematical methodologies have been explored in the past in an effort to quantitatively explain the benefit–risk assessment conclusions and/or to explore a working model tool. These include probabilistic decision analysis [4], use of spatial planes [5], Bayesian approaches [6], patient preferences [7, 8], incremental net health benefit using simulation data [9], number-needed-to-treat (NNT) and number-needed-to-harm (NNH) [10], and a variety of other quantitative approaches [11]. Some notable studies involved the benefit–risk analysis of cancer-related endpoints of pivotal studies from 20 + products for non-small cell lung cancer [12], multiple myeloma [13], and melanoma [14], and another compared the quantitative profile of new chemical entities that were initially approved but subsequently withdrawn from the market [15]. While many approaches highlighted the features of the product’s benefits and risks and specifically the results of the pivotal studies, none of the reviewed publications factored in the critical contributions of risk management, defined in the BRT, as part of the overall decision-making process.

Our proposed approach was to develop a systematic and transparent method to easily explain the benefit–risk decision-making process for regulatory product approval aligned with FDA’s current methodology. In order to propose this as a process that could be applied broadly and be sustained long term for both regulators and industry sponsors, the following requirements were identified: (1) applicable to any therapeutic area or regulated product feature, (2) applicable to any pre- and post-marketing regulatory lifecycle milestone, (3) practical to reflect rapid and real-world needs for communicating with patients, providers, and non-clinical personnel by minimizing extensive mathematical calculations and assumptions, (4) adaptive to any unforeseen future benefits and risks of drugs, biologics, devices, and combinations, and (5) could be used for comparisons among different product profiles. If these requirements are met, this approach could be used to guide approval decision-making for regulators, provide direction to Sponsors for ongoing development, and/or enable patient or provider to use for evaluating treatment options.

We hypothesized that information from the BRT (that includes all of a product’s key benefits, risks, and risk management activities) could be simplified into a numerical value, called the Net-Benefit Score (NBS) by employing codification, quantification, and the use of simple calculations. If the NBS is positive, a favorable net-benefit would be assumed whereby if the NBS is negative, the risks would outweigh the benefits. These net-benefit values would be used as documentation for how regulatory decisions are made presently. As an alternative to using positive and negative integers, the option of a single Benefit–Risk Ratio (BRR) could prove useful by normalizing with a risk denominator, e.g., > 1.0 for a BRR is favorable, < 1.0 is not. To evaluate this methodology, a retrospective review of FDA approved regulatory decisions that included a BRT was used to estimate a % likelihood for product approval.

## Methods

### Identification of Products for Review

The design of this tool is based upon reverse engineering past regulatory approval decisions. As others have noted, only approved product reviews are publicly available for review. Marketing applications that are lacking or likely not to be approved with a complete response letter may be renegotiated and confidentially stored by regulators or withdrawn by the Sponsor to avoid public assessments. In this instance, only new molecular entities (NMEs) or new biologics were selected for evaluation; these products offer a wider set of unknowns given the lack of post-marketing data at the time of approval decision-making. Therefore, product applications that were generics, biosimilars, and 505(B)2 were not included in the review. To avoid selection bias, an annual listing of NMEs and new biological approvals published by the FDA were cross-matched against final review documentation that included BRT. This resulted in a review of 185 products from all therapeutic areas from 2015 to 2019 [16].

### Development of a Scoring System Database

A customized database was built using a web-based software program called Quickbase (Cambridge, MA) to store captured information and perform basic calculations in support of the Net-Benefit Score (NBS) tool. In order to mirror how regulators make product approval decisions, a quantitative accounting model, similar to managing cash flow, uses terms such as a product’s “gross” benefit and “net” benefit scores to frame the starting and end balance of a product’s positive and negative features.

The benefit assessment entailed reviewing the BRT rows labeled “Analysis of Condition”, “Availability of Treatments”, and “Benefits” and were used to define the NBS benefit parameters of severity and prevalence of the condition, the current availability of other products to gauge unmet medical needs, and the robustness (or durability) of the clinical data (Table 1), respectively. Selection of the appropriate levels were translated into a 3-level corresponding weight scale and each assigned a 5-point value scale capped with a maximum ceiling value to avoid overscoring and overstating the benefit since certain multiples of risks can nullify a product’s acceptability and regulatory approval. The verbatim text, used to codify the information, was also captured within the database to enable future reproducibility.

**Table 1:** Benefit Assessment

Disease Severity	Disease Prevalence	Treatment Availability	Durability of Treatment
Mild (reversible, acute, some QOL impact) Moderate (Potential to progress, not reversible without intervention, QOL impact) Severe (death, life-threatening, chronic)	General population Rare disease/orphan Population subset (select population)	No other treatments exist Some products available (1 to 3 products) Many products available (4 or more, multi-class options)	Consistent/clinically meaningful/substantial evidence Likely (strong, surrogate, non-inferior, semi-robust) Not consistent (moderate surrogate)

The risk assessment entailed reviewing the BRT row labeled “Risk”. Each of the key verbatim adverse events were identified, codified into a common text lexicon, and rated according to its severity and frequency based upon the provided categorical descriptors observed across the many product BRTs (Table 2). In contrast with the 2013 version [17], the 2018 BRT had consolidated the “Risk and Risk Management” into a single section [1] (Tables 3 and 4). However, since each identified risk had its own harm potential as described in the BRT, each risk was separately codified and associated with their respective risk mitigation activities. When no specific risk management was documented, routine pharmacovigilance was coded as a default to reflect basic, real-world regulatory requirements.

**Table 2:** Risk Assessment

Severity	Frequency
Severe (death, life-threatening, Grade 4 or 5) Moderate (potential lasting effect, Grade 3) Mild (reversible, brief, Grade 1 or 2)	High frequency or signal (40.1% to 100%) Medium frequency or signal (5.1% to 40%) Low frequency or signal (0 to 5%)

**Table 3:**
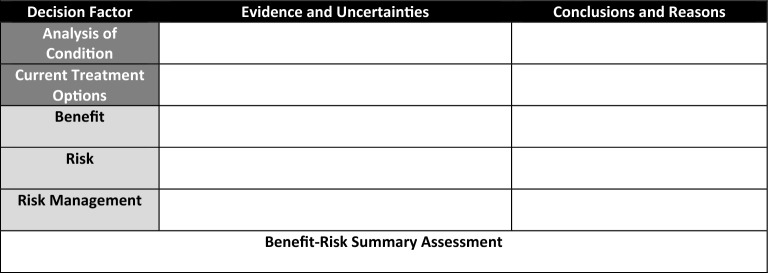
2013 FDA Benefit–Risk Framework

**Table 4:**
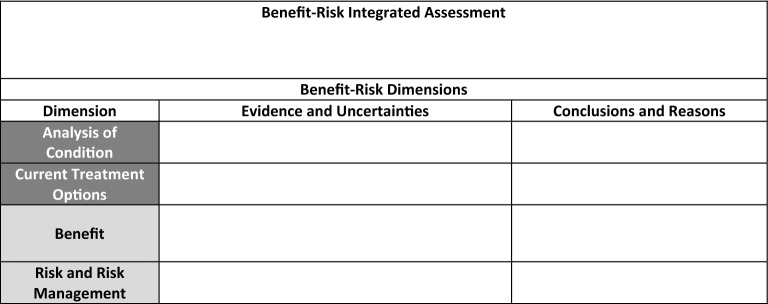
2018 FDA Benefit–Risk Framework

To properly account for the importance of risk management, the mitigation activities were codified and scored according to the relative public health implementation burden to be assumed either by the patient, provider, or Sponsor, e.g., product label highlights in the Warnings and Precautions of a product label involved a low burden, whereas a post-approval pregnancy registry or REMS was scored much higher. Risk mitigation scores were assigned a 5-point scale and capped to avoid overscoring the value relative to the hazard it was intended to prevent.

**Fig. 1:**
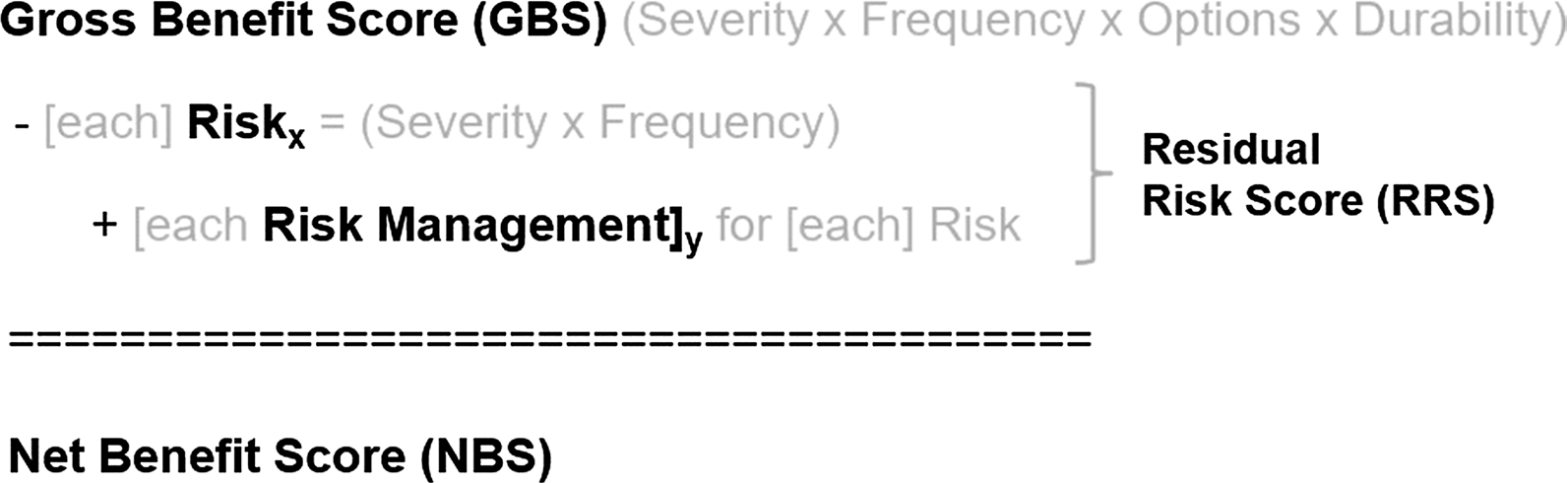
Net-Benefit Score (NBS)

**Fig. 2:**
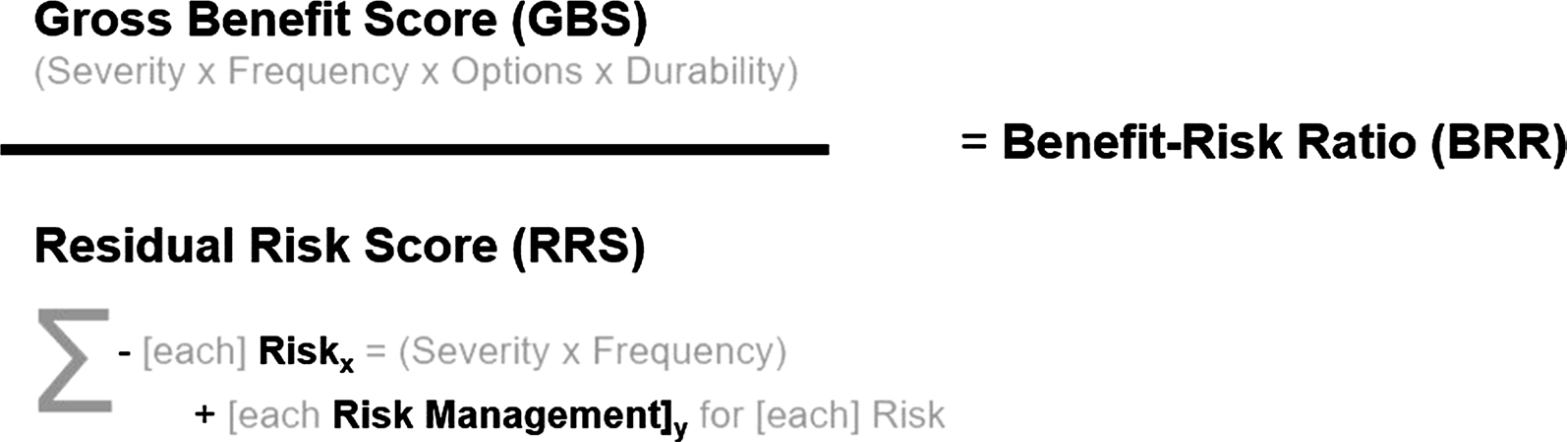
Benefit–Risk Ratio (BRR)

In summary, the NBS is calculated by the sum of the benefits (Gross Benefit Score) minus the sum of each of the risks and the respective risk mitigation activities (lowers the negative impact of the risks which results in a more positive net value) (Residual Risk Score) (Fig. 1). Subsequently, the benefit–risk ratio (BRR) is calculated by dividing the Gross Benefit Score by the Residual Risk Score (Fig. 2).

### Assessors

The scoring of reviewed information within the customized database was performed by a qualified reviewer with a medical background (former regulatory medical officer) and a secondary quality review was performed by a registered pharmacist who presently serves on an institutional review board. Any cross-communication or adjudication from rating differences were documented.

### Review of Information

Over this 5-year period, the types of documents that were available for review evolved from a two- to three-document structure (Summary Review, Clinical Review, Cross-Disciplinary Review) to a single Multidisciplinary Review. In the Benefit–Risk Assessment section of the review, if there was any conflicting information between the BRIA and the BRT, the information in the BRT would be prioritized and utilized for coding and quantification because it contained the most essential “key” factors of risks, benefits, and risk mitigations being considered for final regulatory decision-making. For example, many other risks and benefits may have been highlighted, but may not be important nor relevant to the final decision-making and therefore not scored.

Additional information such as a product’s profile, year of approval, therapeutic area, authorship of reviews by FDA division and office, regulatory designation of any accelerated reviews, e.g., fast-track, etc., were collected during the review. Information associated with chemistry, manufacturing, or control (CMC) issues were considered areas that would need to be resolved before products could be made available to the general public and therefore were not captured. Any current product issues that occurred after the initial regulatory product approval was not codified since no updated BRT was available.

This article does not contain any studies with human or animal subjects performed by any of the authors.

## Results

A total of 220 novel drug and biologic products from the FDA’s approved product listing between January 1, 2015 and December 31, 2019 were identified for review consideration with only 185 products identified with a BRT. Based upon this scoring methodology, all products that have a final positive NBS should be approved. Consequently, instead of an absolute value, a second consideration was to use a BRR approach that would be calculated using the GBS divided by RRS. A BRR of greater than 1.0 would show that product benefits outweigh the risks and therefore would likely to be approved. Table 5 is provided to show any tendencies patterned around each review division.

**Table 5:** Net-Benefit Scores (NBS) and Benefit–Risk Ratio (BRR) by FDA review division

FDA review divisions	# of products	Average Gross Benefit Score (GBS)	Average Residual Risk Score (RRS)	Net-Benefit Score (NBS)	Benefit–Risk Ratio (BRR)
Anesthesia, Analgesia, and Addiction Products (DAAAP)	1	100	− 16	84	6.25
Anti-Infective Products (DAIP)	18	71	− 13	59	9.59
Anti-Viral Products (DAVP)	10	92	− 11	81	19.69
Bone, Reproductive and Urologic Products (DBRUP)	6	67	− 18	49	4.58
Cardiovascular and Renal Products (DCaRP)	5	78	− 7	71	30.86
Dermatology and Dental Products (DDDP)	11	43	− 6	37	12.58
Gastroenterology and Inborn Errors Products (DGIEP)	12	75	− 8	67	14.11
Hematology Oncology Toxicology (DHOT)	1	100	− 31	69	3.23
Hematology Products (DHP)	37	93	− 24	69	13.87
Medical Imaging Products (DMIP)	5	96	− 4	93	54.32
Metabolism and Endocrinology Products (DMEP)	6	80	− 13	68	7.02
Neurology Products (DNP)	23	72	− 16	57	7.79
Oncology Products 1 (DOP1)	14	98	− 29	70	8.80
Oncology Products 2 (DOP2)	13	100	− 19	81	10.40
Psychiatry Products (DPP)	7	78	− 18	60	9.78
Pulmonary, Allergy, and Rheumatology Products (DPARP)	9	72	− 10	62	15.23
Transplant and Ophthalmology Products (DTOP)	7	63	− 6	58	16.44
All products	185	81	− 16	65	13.26

## Discussion

### Reflecting Real-World Use of Benefit–Risk Assessments

The approach for developing a tool to anticipate a regulatory decision outcome was to reverse engineer FDA’s existing and published BRT that has been road tested for the past five years of regulatory product approvals. The aspiration was to develop an adaptive, practical, and universal method to communicate the regulatory decision-making process by accounting for all the benefits, risks, and risk management information of any product in any therapeutic area or lifecycle scenario. In the real world, the mindset of a typical patient/provider relationship is to assess if the benefits of a treatment are reasonable and only then are individual risks considered. Subsequently, once risks are acknowledged, potential mitigations are applied (up to a tolerable threshold for a patient/provider and what expense Sponsors would spend to support) with the intent of ensuring a positive NBS or BRR. This step-by-step consideration for assessing benefit–risk assessment is incorporated into the calculated framework of the NBS and BRR (Table 6).

**Table 6:** Proposed Updated Benefit–Risk Framework

Decision factors		Evidence and Conclusions	Score
Benefit	Condition Severity		
	Condition Prevalence		
	Availability of Options		
	Durability of Results		
Risk	Risk and Mitigations #1		
	Risk and Mitigations #2		
	Risk and Mitigations #x		
Net benefit			

In the real world, regulatory decisions for approval are subject to the inputs of stakeholders (patients, providers, organizations, sponsors, experts, etc.) that may amplify a product’s perceived benefit or risk. None of the reviewed publications included any acknowledgement or integration of risk management into their calculations. Products at face value may have a benefit–risk profile, but insufficient or absent risk mitigation can quickly tip the net-benefit decision of a product. The NBS and BRR attempts to reflect real-world regulatory and patient decision-making by holistically including all three components of benefit, risk, and risk management.

### Availability of Information for Review

The selection of products used to test this hypothesis was determined by reviewing NME and new biologics during the period whereby the BRT had been included in reviews [16]. Since these regulatory products had no prior US-based post-marketing data, examination of these select products would best reflect the most challenging regulatory decisions when only data from controlled clinical studies are available and its impact involves making products available to the general public usually for the first time. During this review, there were known variation in the consistency and formatting of the data and documentation available since these documents, while all from FDA, actually represent the authorship of 17 different drug review divisions and there is no central enforcement of a required BRT format other than from initial training.

### Codification and Scoring

As mentioned earlier, the concept of a Net-Benefit Score or NBS begins with a starting value that is similar to the transactional accounting of gross revenue, called the Gross Benefit Score (GBS), and represents the raw, non-deducted value of the benefit assessment. The Gross Benefit Score (GBS) is calculated using the product of a quantitative equivalent values for the categorical rating of Severity of the Condition (based upon its intended indication), the Prevalence of the Condition, the Availability of Other Treatment Options, and the Durability of the Efficacy Data (statistically significant surrogates vs. clinically meaningfulness) (Fig. 1). When assessing the Durability of Treatment, e.g., double-blind, placebo-controlled study with definitive results with longer duration, are categorically scored better than those data sets that were noted to have only statistical significance and/or used surrogate endpoints.

When performing the risk assessment, the concept of risk analysis involves factoring the likelihood of occurrence against the severity of the harm [18]. Every risk that is identified anywhere in the BRT was codified to a risk term. Each of the risks was subsequently codified by its generally perceived severity (unless modified by descriptive terms) and the frequency of the event. In some products when only non-clinical studies showed a potential safety signal, the frequency of the event was noted as low since the risk may still exist but simply had not been demonstrated in a human population.

The Risk Mitigation Score (RMS) represented the sum total of all the risk mitigation values that were identified in the BRT and had a ceiling value that could not exceed the value of any single risk that it was intended to mitigate. For example, any risk mitigation factor could reduce a risk score to zero; therefore, if a risk was mentioned, a residual risk is still possible unless otherwise described as no risk. The difference of the individual adverse event Risk Score (RS) minus the sum of a capped Risk Mitigation Score represents the Residual Risk Score (RRS), or the risk that may still occur despite all risk mitigation efforts. No additional score value was provided for “Routine pharmacovigilance” when identified as a risk mitigation because all Sponsors are obliged to meet this regulatory requirement.

The Net-Benefit Score (NBS) therefore represents the algebraic sum of these values (GBS-RS + RMS). If the NBS is positive, this would be interpreted as a positive justification for approval whereby benefits outweigh the risks. If the NBS is negative, the risks outweigh the benefit and the product should not be approved. Similarly, the calculations of negative, low, moderate, or high BRR could similarly be deduced. When reviewing the totality of the NBS or the BRR values for each product, the recommended interpretation would simply be categorically “negative”, “marginal (or low)”, “medium”, or “high” net-benefit, versus an emphasis on the exact values given the coarse precision of the coding process.

### Predictability of Regulatory Decision-Making

One application of the NBS and BRR methodology would be to predict the outcome of a regulatory decision-making, e.g., approve or remove an approved product. During pre-approval, while benefit–risk tables are drafted in Development Safety Update Reports or other annual reporting mechanisms, a model using these score parameters could be included in the correspondence and mapped to develop an “approval likelihood” trajectory. Furthermore, after products have been approved, higher NBS and BRR values would suggest a greater margin for tolerating unexpected adverse events before a Sponsor’s product would be considered for removal.

The initial goal for developing the NBS as a single-value, easily interpretable scoring system, was to provide guidance to regulators a rational, repeatable, and logical “net” assessment for decision-making after all benefits and risks have been considered. Consequently, during the development of the final scoring system, we resolved that the proposed approach should reflect past regulatory decisions, e.g., all products should have a positive NBS value and > 1.0 BRR because they were all approved. In order for the tool to be near 100% accurate for prediction, e.g.,a ceiling value for benefits and products with orphan status were weight adjusted. As an example, a product could have an excessive gross benefit value but patients may also only tolerate a level of risk that is too uncomfortable regardless of how much benefit exists. Therefore, a maximum ceiling value for benefit was included in the scoring and orphan status products were given an emphasized value.

### Scoring Patterns

Drawing conclusions across all different review divisions and therapeutic areas is challenging given so many confounders, e.g., any universal approach to assess benefit–risk scores would need to take into account products intended to improve quality of life dermatology ingredients to life-saving emergency treatments. However, some scoring patterns were notable.

#### Interpretation of Values

Individual rater and coding variations are possible and requests were made to make categories more precise for interpretation and reproducibility. Therefore, it would not be appropriate to overly infer the importance of a products’ exact NBS or BRR, but instead to generalize the scores as High, Medium, or Low. Since this involved a retrospective review of products that were already approved, a few products resulted in a low NBS or a BRR near 1.0. We questioned whether such findings would suggest to regulators the need to revisit or increase the vigilance for such products in the post-marketing setting. Similarly, a Sponsor could quickly debunk the scoring system if it was found to be highly unfavorable for a post-approved product that demonstrated continual safety with more patient exposure and experience. At this early research stage, our priorities are to build a method that is scientifically sound and to correctly calibrate the weights for each parameter.

#### Indication

A review by indication has allowed us to visualize quickly on a single scatter plot graph all the compounds by their NBS and BRR. While actual active-control, double-blinded, dummy-blinded, comparator clinical studies should be the scientific standard for treatment comparisons, such studies may not always be feasible to conduct. The NBS and BRR values for products with the same indication and intended use could be selectively used to view the view the landscape of the different approved treatment options for patients, providers, payors, and sponsors.

#### Review Divisions and Offices

Individual FDA regulatory divisions and offices are led by a single director and/or deputy who will typically be the final decision-maker for what benefits, risks and risk mitigations are likely to be factored into the overall regulatory decision-making. As a result, the accuracy, content, and format for how benefits, risks, and risk mitigations as described in the BRT may be more homogeneous within the product portfolio of a division or office. However, review divisions do not have control over what NMEs are being submitted by Sponsors; therefore, any conclusions to be drawn for patterns among review divisions is that they likely represent a surrogate to a therapeutic area. A division such as oncology (Mean NBS = 76; Mean BRR = 12.39) will have a starting point of higher benefit scores given the severity and unmet medical needs of their categorical space compared to the dermatology division, (Mean NBS = 37; Mean BRR = 12.58). Some divisions are also responsible for a wider breadth of medical indications than others.

### Limitations

Product reviews from late 2015 to early 2016 did not have a benefit–risk table (35 products); therefore the results may be skewed from their exclusion. The conclusions drawn from a product’s NDA application review is a snapshot in time of the information that was available during review; any recent post-approval benefits and risks are not included. One of the reviewed products referenced only a label warning and precaution in the BRT but recent review of the approved label now includes a “boxed warning” that is not mentioned; in such cases, only the scenario information that was available at the time of the initial application (publicly released review documents for approval) is used. Some medications may be administered only in monitored settings but was not specifically highlighted in the BRT as a risk mitigation effort (some risk mitigations may be underscored).

### Regulatory Considerations

Some regulatory improvements to be considered for better accuracy and precision of score calculation: (1) encourage all division and office directors to migrate to the single “Multidisciplinary Review” format, (2) reassess and include a BRT whenever a formal meeting is requested with regulators and/or submitting interim regulatory product reports, (3) use MedDRA when describing risks in the BRT for consistent coding to review patterns, (4) use a standard risk mitigation ontology for consistent coding and pattern identification, (5) use a centralized team or persons to ensure consistent formatting and content, and (6) update or re-examine BRT for any significant events to support regulatory actions.

### Future Considerations

Verification with additional reviewers combined with feedback from patient stakeholders could broaden and sharpen the tool accuracy as part of the validation process. Additional explorations may include testing the system with supplement submissions, generic products, 505(B)2, medical device and combination products, reassessing risk scoring calculations of only “moderate” and “severe” adverse events, and following products as new post-marketing signals emerge as part of a lifecycle tracking process.

## Conclusions

As regulatory agencies move towards greater acceptance of real-world conditions and wider input from patient stakeholder groups, an improvement in the format, readability, and transparency with an updated benefit–risk assessment could help support these needs. One such way is to translate regulatory decision-making to quantitative values that could be readily accessible and interpretable for the non-expert while serving as a universal metric that could be followed dynamically to reflect new information. In this proposed approach, the single quantifiable values of either the Net-Benefit Score (NBS) or the Benefit–Risk Ratio (BRR), could provide readers a simple, meaningful, and actionable approach to understanding FDA’s regulatory decision-making as a way to translate the scientific expertise of regulators to the general reader. Moreover, we see this initial retrospective as a way to prospectively assess and/or verify how regulators could make decisions, how Sponsors could focus their efforts on optimizing their product profile with a common value metric, and how downstream patient and provider stakeholders could assess products for use comparisons. Much pattern assessment remains ahead to validate these assumptions in real world, prospective conditions, particularly when only a Sponsor’s pre-submission and partial clinical package is available, but this would be a practical starting point for setting expectations.

## References

[1] US Food and Drug Administration (FDA). Benefit–risk assessment in drug regulatory decision-making. Draft PDUFA VI Implementation Plan (FY2018-2022). Mar 30, 2018.

[2] National Institutes of Health (NIH). Grants & Funding. Scoring Guidance. Link: https://grants.nih.gov/grants/policy/review/rev_prep/scoring.htm. Accessed: May 29, 2020.

[3] Colopy MW, Darmaraju CV, He W (2015). Benefit–risk evaluation and decision making: Some practical insights. Thera Innov Reg Sci.

[4] Caster O, Edwards IR (2015). Quantitative benefit–risk assessment of methylprednisolone in multiple sclerosis relapses. BMC Neurol.

[5] Bennett WE (2016). Quantitative risk–benefit analysis of probiotic use for irritable bowel syndrome and inflammatory bowel disease. Drug Saf.

[6] Costa MJ, He W, Jemiai Y (2017). The Case for a Bayesian approach to benefit–risk assessment: overview and future directions. Ther Innov Reg Sci.

[7] Johnson FR, Hauber AB, Ozdemir S, Lynd L (2010). Quantifying women’s stated benefit–risk trade-off preferences for IBS treatment outcomes. Value Health.

[8] Johnson FR, Zhou M (2016). Patient preferences in regulatory benefit–risk assessments: a US perspective. Value Health.

[9] Lynd LD, Najafzadeh M, Colley L (2010). Using the incremental net benefit framework for quantitative benefit–risk analysis in regulatory decision-making—a case study of alosetron in irritable bowel syndrome. Value Health.

[10] Puhan MA, Singh S, Weiss CO, et al. Evaluation of the benefits and harms of aspirin for primary prevention of cardiovascular events: a comparison of quantitative approaches. *AHRPublication* No. 12(14)-EHC149-EF. Updated Feb 2014. 24404631

[11] Guo JJ, Pandey S, Doyle J (2010). A review of quantitative risk–benefit methodologies for assessing drug safety and efficacy—report of the ISPOR risk–benefit management Working Group. Value Health.

[12] Raju GK, Gurumurthi K, Domike R (2016). A benefit–risk analysis approach to capture regulatory decision-making: non-small cell lung cancer. Clin Pharm Ther.

[13] Raju GK, Gurumurthi K, Domike R (2018). A benefit–risk analysis approach to capture regulatory decision-making: multiple myeloma. Clin Pharm Ther.

[14] Raju GK, Gurumurthi K, Domike R (2019). Using a benefit–risk analysis approach to capture regulatory decision making: melanoma. Clin Pharm Ther..

[15] Patriarca PA, Auken RMV, Kebschull SA (2018). Analysis of the risks and benefits of new chemical entities approved by the US Food and Drug Administration (FDA) and subsequently withdrawn from the US market. Ther Innov Reg Sci.

[16] US Food and Drug Administration (FDA). New Molecular Entity (NME) Drug and New Biologic Approvals. Link: https://www.fda.gov/drugs/nda-and-bla-approvals/new-molecular-entity-nme-drug-and-new-biologic-approvals. Last accessed: Jan 20, 2020.

[17] US Food and Drug Administration (FDA). Structured approach to benefit–risk assessment in drug regulatory decision-making. Draft PDUFA V Implementation Plan. Fiscal Years 2013–2017. February 2013.

[18] US Food and Drug Administration (FDA). ICH Q9 Quality Risk Management. Guidance for Industry. June 2006.

